# Health extension program factors, frequency of household visits and being model households, improved utilization of basic health services in Ethiopia

**DOI:** 10.1186/1472-6963-14-156

**Published:** 2014-04-05

**Authors:** Mezgebu Yitayal, Yemane Berhane, Alemayehu Worku, Yigzaw Kebede

**Affiliations:** 1 University of Gondar, Gondar, Ethiopia; 2 Addis Continental Institute of Public Health, Addis Ababa, Ethiopia; 3 Addis Ababa University, Addis Ababa, Ethiopia; 4 Institute of Public Health, College of Medicine and Health Sciences, University of Gondar, P.O. Box 196, Gondar, Ethiopia

**Keywords:** Health extension program, Health service utilization, Utilization of health extension program

## Abstract

**Background:**

Ethiopia has implemented a nationwide primary health program (the Health Extension Program) at the grassroots level since 2003. The aim of the program is to increase public access to basic health services, mainly by producing model households. These are households which attend at least 75% of the training given by health extension workers and implement at least 75% of the Health Extension Program packages. This study was conducted to assess the extent of the Health Extension Program utilization by the community, and to identify factors associated with it.

**Methods:**

A community-based cross-sectional study was conducted to assess the utilization of the health extension program. Data were collected from 1320 mothers using a structured questionnaire. Multilevel logistic regression was used to identify factors associated with the utilization of the program.

**Result:**

Health extension workers conducted frequent visits to 52.7% (95% CI = 50.0 to 55.4%) of the households, and 78.5% (95% CI = 76.2 to 80.7%) mothers visited health posts. Mothers who had frequent household visits by health extension workers were 1.289 more likely to visit the health posts (AOR = 1.289, 95% CI = 1.028 to1.826) than mothers who did not get frequent visits. Mothers from model households (3 years after graduation) were 2.410 times more likely to visit health post (AOR = 2.150, 95% CI = 1.058 to 4.365) compared to mothers from non-model households. Mothers who felt that they understood the Health Extension Program packages were 1.573 times more likely to visit the health posts (AOR = 1.573, 95% CI = 1.056 to 2.343) than mothers who did not feel they understood the program packages. Mothers from higher income families were 2.867 times more likely to visit health posts (AOR = 2.867, 95% CI = 1.630 to 5.040) compared to mothers from lower income families.

**Conclusions:**

Conducting continuous home visits of non-model households and following up the existing model households, producing more model households by giving model-family training to non-model households, and strengthening the information, education, and communication package are crucial in the implementation of the HEP to increase basic health services utilization.

## Background

Since 2003 the Government of Ethiopia has been implementing the Health Extension Program (HEP) which is an innovative approach initiated to meet the Millennium Development Goals (MDGs) [1]. HEP is designed to provide basic health care to an approximately 5000 people through a health post (HP). Every HP is staffed by two female health extension workers (HEWs) trained for one year and paid directly by the government [2,3].

The HEP has 16 packages of preventive and curative health services that include 3 packages under disease prevention and control (HIV/AIDS and tuberculosis; malaria prevention and control; and first aid emergency measures); 5 packages under family health (maternal and child health, family planning, immunization, nutrition, and adolescent reproductive health); 7 packages under hygiene and environmental sanitation (excreta disposal, solid and liquid waste disposal, water supply and safety measures, food hygiene and safety measures, healthy home environment, control of insects and rodents, and personal hygiene); and 1 package of health education and communication [2,3].

Health service utilization is a result of multiple factors, such as health workers’ conduct, characteristics of the community (family characteristics, social structure, assets/affordability, and perceptions about modern health services). It is also influenced by enabling factors, such as the availability of health facilities, the accessibility to health services, the quality of services, and costs as well as the characteristics of disorder (intensity of illness and number of spells) [4-11]. According to Andersen, factors associated with the utilization of health services can be grouped into predisposing, enabling, and needs factors [12]. In addition, health service characteristics, such as type (hospital, physician, drugs and, medications, or other services); purpose (primary, secondary, or tertiary care), and unit of analysis (contact, volume or episode); health services provision, such as intensity (amount ) and duration (short or long); and frequency of house visits by health workers can have their own effect on health service utilization [13,14].

Health service utilization studies in Ethiopia indicate that factors, such as awareness and perception, literacy, family size, educational status, perceived illness, family income, media exposure, perception of distance to health facilities, perceived transport, and treatment costs are some of the predictor factors [15-19]. The HEP is designed to increase the coverage of primary health care services in Ethiopia, mainly by producing model households using model-family training. The model family training comprises a total of 96 hours of training on basic hygiene and environmental sanitation (30 hours), family health care (42 hours), and disease prevention and control (24 hours). Households which attend at least 75% of the training and implement at least 75% of the HEP packages receive certificates of completion at a graduation ceremony and graduate as model households (families). The program also addresses health service utilization through the establishment of HP to serve 5000 people, and the deployment of two HEWs who conduct home visits in the community and give basic health services in each HP [2].

During the HEP implementation period, particularly from 2007 to 2011, the basic health services indicators such as, contraceptive acceptance (34.8 to 61.7%), antenatal care (52.1to 82.2%), and full immunization (56.4 to 74.5%) have indicated marked improvements. In 2011, in Amhara Region, the rates were 88.6% for contraceptive acceptance, 86.2% for antenatal care, and 82.5% for full immunization [20]. Though there are promising results of the HEP, conducting more studies on assessing the process and the effect has a paramount importance for its successful implementation. Therefore, this study was conducted with the aim of assessing the utilization of the HEP by the community and factors that might be associated with it.

## Methods

### Study design and area

A community-based cross-sectional survey was conducted among women in West Gojjam Zone, Amhara National Regional State, Ethiopia, over a period of three months (March to May, 2012) to assess the utilization of HEP and factors associated with it. West Gojjam Zone was one of the 11 zones of Amhara Region. The HEP was started in 2003, and during the study period, there were 6,530 HEWs (6,401 in rural and 129 in urban areas) of the region, and 782 HEWs (772 in rural and 10 in urban areas) of West Gojjam Zone.

### Study population and sampling

Women who were alive, aged 15–49 years, and had at least one child less than 5 years old were included in the study. A multistage sampling procedure was used to select the study units (mothers in the selected households). At the first stage, six districts (Yilmana Densa, Mecha, Semien Achefer, Bure, Jabi Tehnan and Dega Damot) were randomly selected out of the 13 rural districts. At the second stage, 44 kebeles were selected randomly from the six districts. In the third stage, 30 households were selected randomly from each kebele to get 1320 mothers required for the study. The list of women who were alive, aged 15–49 years, and had at least one child less than 5 years of age in each kebele was prepared in collaboration with the HEWs. Whenever there was more than one mother in a household, only one mother who was responsible for the household was selected randomly by lottery method.

The sample size was determined by using a single population proportion formula. The computation was based on the 95% confidence interval (Z_α/2_ = 1.96), 5% marginal error (d), and 50% utilization of HEP (p) by the community, and a 10% non-response rate.

$$n=\frac{{Z_{\alpha /2}}^{2}p\;\left(1‒p\right)}{d^{2}}=\frac{{\left(1.96\right)}^{2}\left(0.50\right)\;\left(0.50\right)\;}{{\left(0.05\right)}^{2}}=384$$

$$\begin{array}{c} \mathrm{By}\;\mathrm{adding}\;10\%,n=384+10/100\left(384\right)\\ \;=423\;\mathrm{households} \end{array}$$

The sample, 423, was multiplied by the design effect of 3 (number of stages), and the final sample size was 1269, which was again, raised to 1320 in order to take 30 households from each kebele.

### Data collection

In order to collect information, interview was conducted based on a structured questionnaire consisting mainly of close-ended questions. The questionnaire was developed based on previous health service utilization studies carried out in developing countries [11,18,21]. The questionnaire was based on three factors, such as predisposing factors, enabling factors, and needs factors.

Twelve data collectors and six supervisors were recruited and trained to administer the questionnaire. The data collectors were diploma holder nurses and health officers (with a bachelorette degree) served as field supervisors. The interview was conducted at study participants’ houses, and the data collectors had to go house to house in each selected kebele until a sample of 30 mothers was obtained for each kebele.

The structured questionnaire was pre-tested in kebeles of the same administrative zone which were not included in the actual study. The pre-test was done on 66 mothers (5% of the study sample size), and the questionnaire was assessed for its completeness, clarity, and length. A daily data quality-check was done during the study period, and data were double-entered to minimize error during data processing.

### Operational definitions

Utilization of HEP was defined as health post visits by mothers in the community in the last 12 months for health services, such as immunization, family planning, antenatal care (ANC), delivery, postnatal care (PNC), and diagnostic treatment.

Predisposing factors were any demographic (sex, age, marital status), social structure (education, occupation, family size, ethnicity, religion), HEP related (hearing and understanding HEP), and belief related factors (perception about health services and health workers) explaining an individual’s decision to use health services. Enabling factors were situations that influence utilisation behaviour (income, home visits by HEWs, and frequency of home visits, services provided in the HP). Need factors were individuals’ perceived need to use heath care services based on their perceived illness and/or clinical evaluated illness (type and stage of illness).

Model households were defined as households that attended at least 75% of the training given by HEWs and implemented at least 75% of the HEP packages. Frequency of home visits by HEWs was the number of visits HEWs made in a week or month, and we categorized it into more frequent visits if there was at least one visit per month, and no visits or less frequent visits if there was no visit at all or if there was one visit in more than one month. The number of years after household graduation means years since the household has become a model family by attending at least 75% of the HEP training and implementing at least 75% of the HEP packages.

The conduct of HEWs and the quality of services were measured according to study participants’ perception of HEWs’ conduct and services provided in the HP, respectively, using a 5-points Likert scale ranging from 1 (very bad) to 5 (very good). Comprehension/understanding of the HEP was also measured according to study participants’ perception of their HEP knowledge using “Yes” or “No” responses and study participants who mentioned the exact number of HEP packages, i.e.16 packages, were labelled as ‘accurately mentioned the number of HEP packages’.

### Data analysis

Data were coded and entered into Epi-Info 3.5.1 and transferred to SPSS 16 for analysis. Age was coded as “0” for ages below the mean, and “1” for ages above the mean. Mothers’ occupation was coded as “0” for housewife and “1” for farmer and other jobs. Other jobs meant jobs other than housewife and farmer, and included merchant and daily labourer. Education was coded as “0” for illiterate and “1” for at least read and write; income was coded into four categories (0 = Birr 562 and below, 1 = Birr 563–760, 2 = Birr 761–960, and 3 = Birr 961 and above).

The multilevel binary logistic regression was used to assess the predictors of HP visits by the community. The multilevel models allowed us to consider the individual level (household) and the group level (kebele) in the same analysis, rather than having to choose one or the other. Due to the multistage cluster sampling procedure, individual women were nested within kebeles; hence, the likelihood of women seeking HP visits was likely to correlate to the kebele members.

We examined the effect of the individual level variables, and the kebeles using a two-level binary logistic regression modelling. During analysis, the characteristics of women and households were taken as individual level (level-1), and kebeles were treated as level-2. For the dependent variable, HP visit, two models were estimated: the intercept-only model, an empty model that contained no covariates, and the full model that included individual variables and the kebeles. The intercept-only model allowed us to evaluate the extent of cluster variation on the utilization of HEP. Based on this model, the intra-class correlation coefficient (Rho) was calculated to evaluate whether the variation in the scores was primarily within or between clusters.

Both the crude odds ratio (COR) and the adjusted odds ratio (AOR) were used for reporting the results of the binary logistic regression. COR was an unadjusted crude ratio that reported the odds ratio without taking confounders into considerations, while AOR reported the odds ratio taking into account and controlling confounders. Therefore, AOR gave a more accurate picture of the association than COR did. Estimates of population parameters were presented with 95% Confidence Interval (CI). In every application of inferential statistics, *P* value of 0.05 was taken as significance level.

#### *Ethical issues*

The University of Gondar Ethics Review Committee approved the research proposal. A written, informed consent was obtained from each study participant. Confidentiality was assured by not taking personal identifiers. The respondents were also informed about their freedom to withdraw at any time while they were being interviewed.

## Results

A total of 1318 women participated in the study with a response rate of 99.85%, and 1034 (78.5%, 95% CI = 76.2 to 80.7%) study participants visited HPs during the year.

### Predisposing factor

The age of the respondents ranged from 18 to 48 years with a mean age of 32.53 ± 6.25 years. Most of the study participants (90.1%, 95% CI = 88.4 to 91.7%) were married, (74.2%, 95% CI = 71.8 to 76.6%) illiterate, (79.0%, 95% CI = 76.8 to 81.2%) housewives, 100% Orthodox Christians, and 99.3% Amhara. The average family size was 5.53 ± 1.79 individuals (Table 1).

**Table 1:** **Socio**-**demographic characteristics of study participants in West Gojjam Zone**, **Ethiopia**, **2012**

**Variables**	**Number**	**Percent (95% CI)**
***Age***		
24 and below	115	8.8 (7.2 - 10.3)
25 - 34	659	50.3 (47.6 - 53.1)
35 and above	535	40.9 (38.2 - 43.5)
Total	1309	100
***Marital status***		
Single, separated, widow or divorced	131	9.9 (8.3 - 11.6)
Married	1187	90.1 (88.4 - 91.7)
Total	1318	100
***Education***		
Illiterate	978	74.2 (71.8 - 76.6)
At least read and write	340	25.8 (23.4 - 28.2)
Total	1318	100
***Occupation***		
Housewife	1041	79.0 (76.8 - 81.2)
Farmer and other jobs	277	21.0 (18.8 -23.2)
Total	1318	100
***Family size***		
2-3	162	12.7 (10.9 - 14.5)
4-5	495	38.9 (36.2 - 41.5)
6-7	435	34.1 (31.5 - 36.8)
8 and above	182	14.3 (12.4 – 16.2)
Total	1274	100

The majority, 1211 (91.9%, 95% CI = 90.4 to 93.4%) of the study participants had heard about the HEP, and the HEWs were the source of information for 1199 (91.0%, 95% CI = 89.4 - 92.5%) of them. Out of the 1211 study participants, 890 felt that they understood/comprehended what the HEP was. Packages such as Immunization, Excreta Disposal, Family Planning, and Solid and Liquid Wastes were mentioned by 840, 828, 827, 814 study participants, respectively. On the contrary, the least recalled packages, such as First Aid, Rodent and Insect Control, Adolescent Reproductive Health and Nutrition were mentioned by 196, 313, 347, and 396 study participants, respectively. The conduct of the HEWs was rated as “Good” by 94.7% (95% CI = 93.5 to 96.0%) of the participants. Similarly, the services provided in the HPs were viewed of “Good Quality” by 94.4% (95% CI = 93.1 to 95.6%) of them (Table 2).

**Table 2:** **Knowledge and preferences of study participants about health extension program in West Gojjam Zone**, **ANRS**, **Ethiopia**, **2012**

**Variables**	**Number**	**Percent (95% CI)**
**Heard about HEP (1318)**		
No	107	8.1 (6.6 - 9.6)
Yes	1211	91.9 (90.4 - 93.4)
Total	1318	100
***Sources of information***		
HEWs	1190	91.0 (89.4 - 92.5)
Other Health Workers	251	19.0 (16.9 - 21.2)
Community	217	16.4 (14.4 - 18.4)
Radio	56	4.2 (3.2 - 5.3)
Total	1318	1318
***Understanding HEP components***		
No	428	32.5 (30.0 - 35.0)
Yes	890	67.5 (65.0 - 70)
Total	1318	100
***Which HEP do you know? (890)***		
Immunization	840	63.7 (61.1 - 66.3)
Excreta disposal	828	62.8 (60.2 - 65.4)
Family planning	827	62.7 (60.1 - 65.4)
Solid and liquid waste disposal	813	61.8 (59.1 - 64.4)
Food supply and safety measures	795	60.3 (57.7 - 63.0)
Personal hygiene	785	59.6 (56.9 - 62.2)
Water supply and safety measures	741	56.2 (53.5 -58.9)
Health house environment	737	55.9 (53.2 - 58.6)
Malaria	694	52.7 (50.0 -55.4)
Maternal and child health	572	43.4 (40.7 - 46.1)
HIV/AIDS, Other STD and TB	455	34.5 (32.0 - 37.1)
Nutrition	396	30.0 (27.6 - 32.5)
Adolescent reproductive health	347	26.3 (23.9 - 28.7)
Insect and rodent control	313	23.7 (21.4 - 26.0)
First aid	196	14.9 (12.9 - 16.8)
Total	1318	100
***Health institution preferences (visits) by the community***		
Health post	910	69.0 (66.5 - 71.5)
Health center	926	70.3 (67.8 - 72.7)
Hospital	31	2.4 (1.5 - 3.2)
Private health facility	112	8.5 (7.7 – 10.0)
Total	1318	100
***Community perception about the conduct of HEWs***		
Bad	70	5.3 (4.1 - 6.5)
Good	1248	94.7 (93.5 - 96.0)
Total	1318	100
***Community perception about the quality of services in the HP***		
Bad	74	5.6 (4.4 - 6.9)
Good	1244	94.4 (93.1 -95.6)
Total	1318	100

### Enabling factors

The average monthly income of the households was Birr 888 ± 588. One thousand six (76.3%, 95% CI = 74.0 to 78.6%) households were model households. Only 3.0% (95% CI = 2.0 to 3.9%) of the study participants had roles as leaders or secretaries of committees in the community or had government employment. One thousand one hundred ten study participants (84.2%, 95% CI = 82.2 - 86.2%) had home visits by HEWs, and 52.7% (95% CI = 50.0 - 55.4%) had frequent visits (at least one visit every 4 weeks). Most of the study participants (98.5%) travelled on foot to visit the health posts (Table 3).

**Table 3:** **Study participants**’ **income**, **social**, **and health extension program related status in West Gojjam Zone**, **Ethiopia**, **2012**

**Variables**	**Number**	**Percent (95% CI)**
***Family monthly income (Eth. Birr)***		
562 and below	331	25.1 (22.8 - 27.5)
563-760	330	25.0 (22.7 - 27.4)
711-960	353	26.8 (24.4 - 29.2)
961 and above	304	23.1(20.8 - 25.3)
Total	1318	100
***Graduated household***		
No	312	23.7 (21.4 - 26.0)
Yes	1006	76.3 (74.0 - 78.6)
Total	1318	100
***Years after graduation (became model households)***		
Not graduated	312	23.7 (21.4 - 26.0)
1-2 years	636	48.2 (45.6 -51.0)
3 years	238	18.1(16.0 - 20.1)
4-5 years	132	10.0 (8.4 - 11.6)
Total	1318	100
***Status in the community***		
Ordinary member	1279	97.0 (96.1 - 98.0)
Committee member, community leader or government employee	39	3.0 (2.0 - 3.9)
Total	1318	100
***Home visits by HEWs***		
No	208	15.8 (13.8 - 17.8)
Yes	1110	84.2 (82.2 - 86.2)
Total	1318	100
***Frequency of home visits by HEWs***		
No visits or less frequent visits	623	47.3 (44.6 - 50.0)
More frequent visits (at least one visit every 4 weeks)	695	52.7 (50.0 - 55.4)
Total	1318	100
***Means of transportation for study participants while visiting HP***		
On foot	1018	98.5 (97.7 - 99.2)
On horse/mule back	7	0.7 (0.2 -1.2)
By car	9	0.8 (0.3 - 1.4)
Total	1034	100

### Needs factors

The main reasons for visiting HPs were family planning, immunization, and child illness. In cases of illness, 55.6% (95% CI = 52.9 - 58.3%)of the study participants visited the HP soon after the illness began, and 21.5% (95% CI = 19.3 - 23.7%) only if there was no improvement (Table 4).

**Table 4:** **Reasons and stage of illness for seeking health post visits in West Gojjam Zone**, **Ethiopia**, **2012**

**Variables**	**Number**	**Percent (95% CI)**
***Reasons for visiting the HP***		
Family planning	859	65.2 (62.6 - 67.7)
Immunization	830	63.0 (60.4 - 65.6)
Child illness	788	59.8 (57.1 - 62.4)
Family illness	482	36.6 (34.0 - 39.2)
Antenatal	278	21.1 (18.9 - 23.3)
Delivery	129	9.8 (8.2 - 11.4)
Postnatal care	116	8.8 ( 7.3 - 10.3)
Total	1318	100
***At what stage of the illness do you visit the HP?***		
Soon after the illness starts	733	55.6 (52.9 - 58.3)
No improvement of the illness	283	21.5 (19.3 - 23.7)
If the sick person unable to eat/drink	13	1.0 (0.5 -1.5)
Other	5	0.4 (0.05 – 0.7)
Total	1318	100

### Multilevel logistic regression

The bivariate logistic regression showed that HP visits had significant association with mothers’ occupation, family income, hearing (having information) about HEP, understanding the HEP, perception about the quality of services provided in the HP, perception about the conduct of HEWs, frequency of home visits by the HEWs, and household graduation status. However, HP visits had no significant association with age, marital status, educational status, ethnicity, religion, family size, status in the community, and means of transportation to visit the HP. In the multilevel logistic regression, hearing about HEP, perception about the health services provided in the HP and the conduct of HEWs, and mothers’ occupational status were not found significant. Household graduation status, frequency of home visits by HEWs, understanding the HEP and family income were predictors of HP visits by the community.

Mothers who had frequent household visits by the HEWs were 1.289 more likely to visit the HP (AOR = 1.289, 95% CI = 1.028 to1.826) than mothers who did not have frequent household visits. Mothers from model households (3 years after graduation) were 2.150 times more likely to visit the HP (AOR = 2.150, 95% CI = 1.058 to 4.365) compared to mothers from non-model households. Mothers who felt that they understand the HEP packages were 1.573 times more likely to visit the HP (AOR = 1.573, 95% CI = 1.056 to 2.343) than mothers who did not feel they understood the program packages. Mothers from higher income families were 2.867 times more likely to visit the HP (AOR = 2.867, 95% CI = 1.630 to 5.040) compared to mothers from lower income families (Table 5).

**Table 5:** **Factors Associated with utilization of HEP by the community**, **West Gojjam Zone**, **Ethiopia**, **2012** (**n** = **1318**)

***Variables***	***Utilization (HP visits)***		***Crude OR (95% CI)***	***Adjusted OR (95% CI)***
	**Yes**	**No**		
**Mothers’ occupation**				
Housewife	837	204	1.0	1.0
Farmers and others	197	80	0.600 (0.444, 0.811)	0.920 (0.494, 1.715)
**Family Income**				
562 and below	228	103	1.0	1.0
563-760	253	77	1.484 (1.051, 2.096)	1.189 (0.774, 1.827)
761-960	277	76	1.647 (1.167, 2.323)	1.275 (0.817, 1.989)
961 and above	276	28	4.453 (2.831, 7.005)	3.867 (1.630, 5.040)*
**Heard about HEP**				
No	51	56	1.0	1.0
Yes	983	228	4.734 (3.155, 7.104)	1.198 (.604, 2.377)
**Understanding HEP**				
No	284	144	1.0	1.0
Yes	750	140	2.716 (2.074, 3.557)	1.573 (1.056, 2.343)**
**Perception on quality of services in HP**				
Bad	35	39	1.0	1.0
Good	999	245	4.544 (2.819, 7.323)	0.634 (0.356, 1.127)
**Perception about the conduct of HEWs**				
Bad	38	32	1.0	1.0
Good	996	252	3.328 (2.039, 5.434)	0.804 (0.455, 1.421)
**Frequency of home visits**				
No visits or less frequent visits	450	173	1.0	1.0
More frequent visits	584	111	2.023 (1.547, 2.645)	1.289 (1.028, 1.826)**
**Household graduation status**				
Not graduated	203	109	1.0	1.0
1-2 years s	509	127	2.152 (1.589, 2.914)	1.310 (0.780, 1.830)
3 years	212	26	4.378 (2.739, 6.999)	2.150 (1.058, 4.365)**
4-5 years	110	22	2.685 (1.607, 4.487)	1.666 (0.736, 3.771)

*p<0.01, **p < 0.05.

## Discussion

The study showed that the number of years since a household graduated, frequency of HEWs household visits, understanding the components of HEP packages, and having higher family income were predictors of HP visits by the Community. However, mothers’ occupational status, hearing about HEP packages, perception of the community about the quality of services provided in the HP and the conduct of HEWs were not predictors of HP visits.

The frequency of HEWs household visits and the number of years after household graduation had a significant positive association with HP visits by the community. The findings regarding the effect of number of visits on the outcomes of home visit programs, such as the utilization of health services were mixed [22]. A meta-analysis of home visiting programs for at-risk families to examine differences in the effects of programs on maternal behaviour, noted that the effectiveness of home visit programs was principally dependent upon the frequency of services, stating that programs with more frequent contact between home visitors and their clients were most successful [23]. The same study suggested that the duration might be less important than the frequency of services, noting that short-term intensive interventions could be highly effective. In contrast to these findings, some systematic reviews of home visiting programs found no pattern of difference in the average intensity and duration of the program related to the outcomes measured [24,25].

Understanding the components of the HEP had a significant positive association with the utilization of the HEP. This was consistent with studies done in Taiwan and Uganda that identified individual and community knowledge and acceptance of health services or health literacy had a significant positive association with health service utilization [18,26]. Immunization, excreta disposal, family planning, solid and liquid waste disposal were packages most recalled by the participants, while first aid, insect and rodent control, adolescent reproductive health and nutrition were packages least recalled. This may be due to the focus of the HEWs on packages such as immunization and family planning which are now becoming more accepted by the community, and packages such as excreta disposal, and solid and liquid waste disposal which are considered as good signs of the HEP implementation by the community.

In this study, higher family income (Birr 961 and above) had a positive association with the HP visits by the community, which was consistent with the health service utilization studies in Uganda and Bangladesh that reported income as one of the important factors in overcoming barriers to utilization of health services [8,11,14,21].

The study indicated that mothers’ occupation had no significant association with HP visits. This was consistent with a study on correlates of and barriers to the utilization of health services for delivery in South Asia and sub-Saharan Africa which reported the odds of using a health facility for delivery were about the same for both working and non-working women in Tanzania, Bangladesh, India and Pakistan [27]. However, studies conducted in Nepal indicated that nonworking women were more likely to receive maternal health services than working women and reasoned out that most women in Nepal were working for family survival in the agriculture sector [28,29]. Another study in Iran also revealed that housewives were more likely to seek outpatient care than other job holders [30].

The perception of the community about the conduct of HEWs, and the quality of services provided in the HP had no significant association with the HP visits. Although there was good perception about the quality of the services provided in the HPs by the majority of the study participants, lack of drugs and supplies, inability of the HEWs to diagnose, and poor services were the reasons mentioned by the other participants for having bad perception about the quality of services in the HPs. A study in India on the quality of healthcare services indicated that quality issues, such as healthcare delivery, health personnel conduct, and drug availability significantly impacted on the perception of women influencing the utilization of health services [4].

We also assessed the reasons for visiting HPs or consulting HEWs. Accordingly, family planning, immunization, child illness, and family illness were the most mentioned reasons while ANC, delivery, and PNC services were the least mentioned for visiting HPs and consulting HEWs. This might be due to the community perception that services like immunization and family planning could be provided at the HP level by HEWs and services like ANC, delivery, and PNC were better provided at health centers and hospitals by nurses or doctors. But this needs further investigation. In case of illness, most of the participants visited the HPs soon after the illness started, but still there were participants who visited the HP only if there was no improvement of the illness. This was consistent with previous studies that indicated that perceived morbidity and the presence of disabling health problem were predictors of health service utilization [16,18].

### Limitations

Though the study was based on a large sample with a high response rate due to our effort to include 30 households from each kebele, it had the following limitations. Firstly, this cross sectional study provided only a snapshot (one point in time) of utilization of the HP visits by the community, and we could not ascertain a causal relationship but rather tendencies and associations. Secondly, recall bias was expected in this study since the study participants might not remember all the HEP services and associated factors. Lastly, there was lack of baseline information in the study area about basic health services before the implementation of the HEP, and we could not compare the results of this study with base line data.

## Conclusions

The study indicated that utilization of the HEP had a significant positive association with HEP implementation related factors, such as number of years after graduation, frequency of household visits by HEWs, and understanding the HEP. The utilization of the HEP also had a significant positive association with family income. Thus, conducting continuous home visits of non-model households and following up the existing model households, producing more model households by giving model-family training to non-model households, and strengthening the information, education, and communication package are crucial in the implementation of the HEP to increase basic health service utilization.

Unlike the conventional CHWs who train for a few months and get either incentives or per diem for their work, the HEWs get one year training and are full-time employees receiving regular monthly salary from the government to implement the HEP packages. This could trigger a sense of ownership and motivate the HEWs to implement the HEP. Other countries of similar context with Ethiopia may apply the HEP-like health programs that reach the community at the grass-roots level. The association of HEP related factors was evident in this study, but the fact that other factors, such as the availability of voluntary community health workers, agricultural development agents, and health promotion through the mass media could also affect service utilization and should be taken into consideration. Therefore, further studies which use more robust study designs and data analysis methods focusing on specific health services such as ANC and skilled delivery services are required for better understanding of HEP contribution to health services utilization.

## Competing interests

The authors declare that they have no competing interests.

## Authors’ contributions

MY conceived the study, participated in the design, collection, and analysis of the study, and drafted the manuscript. YB participated in the conceptualization and design of the study and helped to draft the manuscript. AW helped in the design of the study and analysis of the data, and contributed to the drafting of the manuscript. YK also participated in the design of the study and contributed to the drafting of the manuscript. All authors read and approved the final manuscript.

## Authors’ information

MY is a lecturer of Health Service Management and Health Economics at the Institute of Public Health, the University of Gondar. He is also a PhD student at the University of Gondar and Addis Continental Institute of Public Health Joint PhD Program doing his dissertation on the implementation of the HEP in West Gojjam Zone, Amhara National Regional State, Ethiopia. YB is a Professor in Epidemiology and Public Health at the Addis Continental Institute of Public Health, Addis Ababa, Ethiopia. AW is Associate Professor of Biostatistics at Addis Ababa University, Addis Ababa, Ethiopia. YK is a Professor in Public Health at the University of Gondar and Director of Dabat Rural Health Research Center at the University, Gondar, Ethiopia.

## Pre-publication history

The pre-publication history for this paper can be accessed here:

http://www.biomedcentral.com/1472-6963/14/156/prepub
